# Olfactory Specialization in *Drosophila suzukii* Supports an Ecological Shift in Host Preference from Rotten to Fresh Fruit

**DOI:** 10.1007/s10886-015-0544-3

**Published:** 2015-01-25

**Authors:** Ian W. Keesey, Markus Knaden, Bill S. Hansson

**Affiliations:** Department of Evolutionary Neuroethology, Max Planck Institute for Chemical Ecology, Hans-Knöll-Str. 8, 07745 Jena, Germany

**Keywords:** Olfaction, Chemical ecology, Neuroethology, Specialization, Drosophila, Insect behavior

## Abstract

It has been demonstrated that *Drosophila suzukii* is capable of attacking ripening fruit, making it a unique species within a fly family named for their attraction towards the fermentation products associated with rotten fruits, vinegar, and yeast. It also has been hypothesized that *D. suzukii* is more attracted to the volatiles associated with the earlier ripening stages of fruit development, and in turn, that *D. suzukii* is less attracted to fermented food resources, especially when compared with *D. melanogaster*. Here, we demonstrate that *D. suzukii* and its close relative *D. biarmipes* are in fact more sensitive to volatiles associated with the fruit-ripening process; however, in choice-assays, both spotted-wing species are more attracted to fermented fruit than to earlier stages of fruit development, which is similar to the behavioral preferences of *D. melanogaster*, and thus, fruit developmental stage alone does not explain the ecological niche observed for *D. suzukii*. In contrast, we show that both *D. suzukii* and *D. biarmipes* are more attracted to leaf odors than *D. melanogaster* in behavioral trials. For *D. suzukii*, this differential behavioral preference towards leaves appears to be linked to β-cyclocitral, a volatile isoprenoid that we show is most likely a novel ligand for the “ab3A” neuron. In addition, this compound is not detected by either of the other two tested fly species.

**Electronic supplementary material:**

The online version of this article (doi:10.1007/s10886-015-0544-3) contains supplementary material, which is available to authorized users.

## Introduction

Like most insects, the members of the genus *Drosophila* rely on olfactory information to follow navigational cues associated with suitable feeding and oviposition sites. These odor cues often are connected to a distinct ecological niche for a particular *Drosophila* species, with subsequent evolutionary adaptations to the olfactory system that further support and enhance the identification of, and navigation towards, these chemically distinct habitats. Several species of *Drosophila* have been studied according to their species-specific neuroethology, including *D. sechellia* (Dekker et al., 2006; Stensmyr et al., 2003), *D. erecta* (Linz et al., 2013), and *D. mojavensis* (Date et al., 2013). However, none have been more extensively examined than *D. melanogaster*, which is the molecular and genetic model for olfactory research (De Bruyne et al., 2001; Hallem and Carlson, 2006; Knaden et al., 2012).

Currently, an outbreak of a new insect, *Drosophila suzukii* (Matsumura) has spread across much of North America (Lee et al. 2011, 2012), as well as Europe (Calabria et al. 2012). This new *Drosophila* species has presented a novel opportunity to advance the integrated pest management (IPM) efforts to control it. In addition, it has provided an opportunity to compare the evolutionary neuroethology that propels one fly species towards world-wide pest status, while the other members of the same genus are not of great agricultural or economic concern. The main reason for *D. suzukii* quickly rising to become a large-scale agricultural problem involves its ability and preference towards attacking and damaging fresh, ripe fruit that is often still attached to the host plant. This is opposed to the model organism, *D. melanogaster*, as well as most of the other studied members of the genus *Drosophila*, which are known to have a preference for overripe, rotten, or fermenting fruit, as well as yeast. In contrast to the other studied *Drosophila* species, the adults of *D. suzukii* inflict economic damage in a wide number of fruit industries, including cherries, raspberries, strawberries, and blueberries. In addition, one of the major morphological adaptations noted for *D. suzukii* is an enlarged and heavily sclerotized ovipositor, which it can use in a saw-like motion to penetrate fresh fruit and insert single eggs below the fruit surface (Atallah et al., 2014).

Several research efforts already have been made to trap and monitor *D. suzukii*, many of which have met with some success by using common fermentation baits, such as components of yeast, vinegar, or wine (Basoalto et al., 2013; Cha et al., 2013; Landolt et al., 2012; Lee et al. 2012); however, none of these trapping studies have identified a trapping system that is more attractive to *D. suzukii* than any of its other similar *Drosophila* relatives, thus making sorting and counting trapped flies difficult if not impossible for those involved in IPM efforts.

Thus, in order to identify important evolutionary shifts in olfaction, the antennae and large basiconic sensillae of *D. suzukii* have been compared to the well-studied *D. melanogaster* olfactory system. Additionally, a third species, *D. biarmipes*, which is the closest relative of *D. suzukii* that has its genome sequenced and that also possesses an understudied ecology, was further selected for the comparison of host preference shifts across this genus. Our research goals here were two-fold, directed first towards understanding the neuroethology that makes these three fly species unique in their host preference, and second, towards enhancing the generation of an effective, species-specific monitoring tool to assist in protecting a diverse array of agricultural ecosystems from economic damage.

## Methods and Materials

### Fly Stocks

Our *D. suzukii* (14023–0311.01) and *D. biarmipes* (14023–0361.10) wild-type flies were both obtained from the UCSD Drosophila Stock Center (www.stockcenter.ucsd.edu). All experiments with wild-type *D. melanogaster* were carried out with Canton-S (stock #1), which were obtained from the Bloomington Drosophila Stock Center (www.flystocks.bio.indiana.edu). Stocks were maintained according to Stokl et al. (2010), and for all experiments we used 2–7 d -old flies of both sexes. No differences were noted between the sexes in regard to physiology or behavior, and thus, the data were pooled.

### Stimuli and Chemical Analysis

All synthetic odorants that were tested were acquired from commercial sources (Sigma, www.sigmaaldrich.com and Bedoukian, www.bedoukian.com) and were of the highest purity available. Stimuli preparation and delivery followed Stokl et al. (2010), and the headspace collection of volatiles was carried out according to standard procedures. GC/MS analyses were performed on all volatile collections as described previously (Stensmyr *et al*., 2012), and NIST mass-spectral library identifications were confirmed with the injection of chemical standards.

### Behavioral Assays and Electrophysiology

Trap experiments were performed as previously described for individual compounds (Date et al., 2013; Knaden et al., 2012), but without pipette tip entrances to the trap (as *D. suzukii* adults were too large to enter) and instead an additional 200 μl of light mineral oil (Sigma-Aldrich, 330779-1 L) was used to capture and drown flies upon entrance to the container. All behavioral traps consisted of 60 ml plastic containers (Rotilabo sterile screw cap, Carl Roth GmbH, EA77.1), with one trap used as a blank control and the other containing the treatment odor. In experiments with whole fruit, each stage was placed individually in traps that were presented simultaneously, and a larger arena was used (http://bugdorm.megaview.com.tw/index.php, BugDorm-44545 F). GC/EAD and GC/SSR measurements were performed as described previously (Stensmyr *et al*., 2012). All dilutions were prepared in hexane, and all behavioral trials were conducted with compounds diluted to 10^−3^ unless otherwise noted. Statistics were performed using GraphPad InStat version 3.10 at both α = 0.05 and α = 0.01 levels. No differences were noted between the sexes in regard to physiology or behavior, and thus, the data were pooled.

## Results

### Assessment and Comparison of Large Basiconic Sensillae

**Fig. 1:**
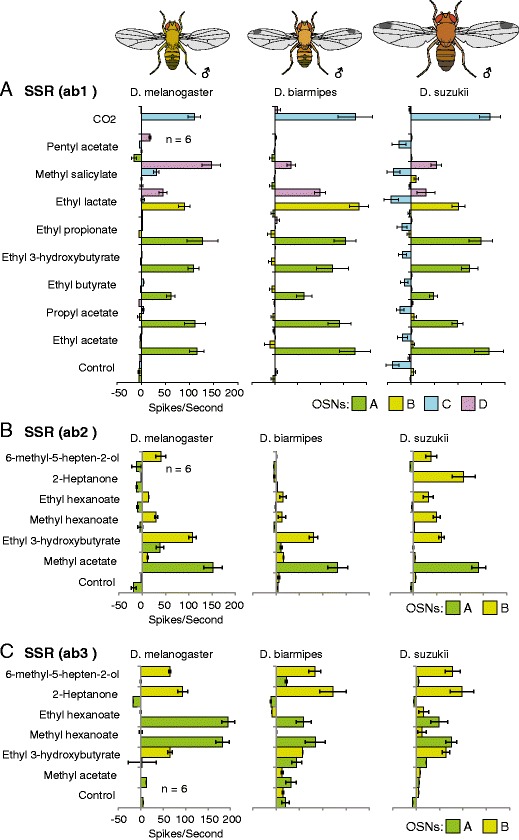
Diagnostic response profiles for the olfactory sensory neurons (OSNs) housed within the large basiconic sensilla of the three *Drosophila* species as measured from single sensillum recordings. Data are presented as spikes per second (+/− SEM), with the largest neuron amplitude being designated A, and B neuron the second largest, and so forth. (**a**) Response profiles of the 4 OSNs housed in the “ab1” neuron. (*Green* = “ab1A”; *Yellow* = “ab1B”; *Blue* = “ab1C”; *Purple* = “ab1D”). (**b**) Response profiles of the 2 OSNs housed in the ab2 neuron. (*Green* = “ab2A”; *Yellow* = “ab2B”). (**c**) Response profiles of the 2 OSNs housed in the ab3 neuron. (*Green* = “ab3A”; *Yellow* = “ab3B”)

In order to successfully navigate and record from the three large basiconic sensilla types, a small panel of diagnostic odors was used to identify each sensillum type across all fly species tested (Fig. 1). The “ab1” sensillum is quite different from the other two large basiconics in that it contains 4 OSNs (olfactory sensillum neurons), as well as demonstrates a consistently strong response to CO2 stimulation; however, we could not detect many response differences among the fly species by using this sensillum type (Fig. 1a). The “ab2” sensillum contains 2 OSNs, with the larger “A” neuron responding stronger to methyl acetate and the smaller “B” neuron responding more strongly to ethyl 3-hydroxybutyrate (Fig. 1b). The response of this sensillum type was quite similar towards the diagnostic odor panel used for each of the 3 species, with the only difference noted in *D. suzukii*, where the “B” neuron also displayed strong responses to 2-heptanone, a response that was not seen in the other two fly species. Lastly, the “ab3” sensillum also contains 2 OSNs, and in *D. melanogaster* the larger “A” neuron responds more strongly to methyl and ethyl hexanoate with the smaller “B” neuron responding more strongly to 2-heptanone and 6-methyl-5-hepten-2-ol (Fig. 1c), a response profile that matches previously reported results for this species. However, while the “B” neuron in *D. biarmipes* and *D. suzukii* was quite similar to *D. melanogaster*, the “ab3A” neuron was noticeably different. More specifically, the “A” neuron within the “ab3” sensillum for both *D. biarmipes* and *D. suzukii* had a markedly reduced response to the fermentation products, ethyl and methyl hexanoate.

### Stages of Fruit Development

**Fig. 2:**
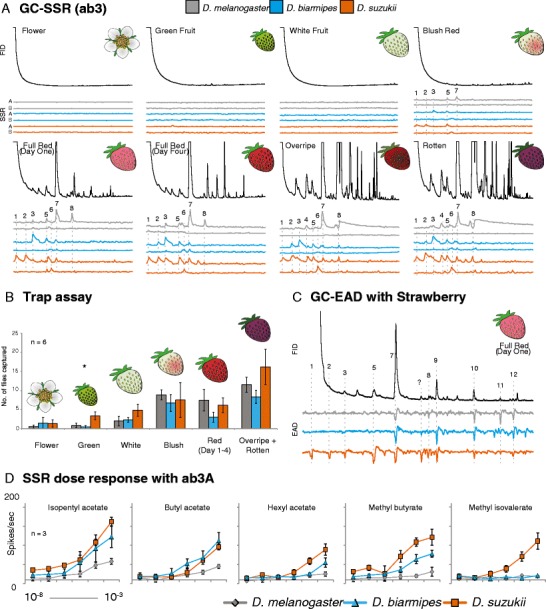
Electrohysiological and behavioral responses using fruit chemistry. (**a**) GC/coupled single sensillum recordings (“ab3” sensillum) with headspace samples from eight distinct stages of fruit development. Headspace collections are shown above (FID) with the respective A and B neuron response for each species shown below (SSR) (*N* = 3). (*Grey* = *Drosophila melanogaster*; *Blue* = *D. biarmipes*; *Orange* = *D. suzukii*). (**b**) Trap-capture rates for the three *Drosophila* species using the stages of strawberry development. An *asterisk* denotes a significant differences (α = 0.05). Only one stage was significantly different among the species (*Green*, *P* = 0.019; two-tailed, paired *t*-test). No other stages were significantly different among the species (*Flower*, *P* = 0.63; *White*, *P* = 0.08; *Blush* = 0.42; *Red*, *P* = 0.12, Rotten, *P* = 0.23). (**c**) GC-couple electroantennogram (GC/EAD) recordings with the headspace of full red strawberry fruit (*N* = 3). GC peaks were identified using GC/MS (and confirmed with synthetic standards) as (*1*) Methyl butyrate, (*2*) methyl isovalerate, (*3*) butyl acetate, (*4*) isopropyl butyrate, (*5*) isopentyl acetate, (*6*) 2-butoxy ethanol, (*7*) methyl hexanaote, (*8*) ethyl hexanoate, (*9*) hexyl acetate, (*10*) linalool, (*11*) benzyl acetate, (*12*) methyl salicylate. (**d**) Dose response curves for the “ab3A” OSN to several compounds identified from strawberry fruit headspace for which *D. suzukii* is more sensitive than *D. melanogaster*

To assess the sensitivity of each of the three *Drosophila* species towards whole, intact and commercially available ripe fruit, we examined the respective GC/EAD responses of each fly species towards the headspace of ripe strawberry (Fig. 2c). Here, we observed that *D. suzukii* was more sensitive than *D. melanogaster* towards several fruit odors, including methyl butyrate, methyl isovalerate, butyl acetate, isopentyl acetate, and hexyl acetate. In order to test the hypotheses that *D. suzukii* detects the ripening fruit earlier and is more attracted to earlier stages of fruit development than the other two species, we generated headspace odor collections from eight distinct stages of development using the traditional garden strawberry (*Fragaria* × *ananassa*), including odor collections from the flowering stage through to rotten fruit (Fig. 2a). These eight volatile odor collections then were used subsequently in GC/EAD and GC/SSR trials for each of the three tested *Drosophila* species (*N* = 3). We could show that *D. suzukii* was more sensitive to several odors associated with ripening strawberries, and moreover, that this difference in antennal sensitivity could be explained largely by the observed differences in responses associated with the “ab2” and “ab3” sensilla types (Fig. 2a, 2D; Supplemental 1A, 1B), while no significant differences were noted across the “ab1” sensilla in GC/SSR trials (data not shown). More specifically, in the case of the “ab2” sensillum, *D. suzukii* appeared to detect the fruit before the other two species, namely during the blush red phase of fruit development (isopentyl acetate; Supplemental 1B). In addition, all three *Drosophila* species first detected the ripening fruit during the blush red phase using the “ab3” sensillum type, although utilizing different chemistry (Fig. 2a). In the case of *D. melanogaster*, isopentyl acetate and methyl hexanoate were first detected (peaks 5 and 7), whereas in *D. biarmipes*, butyl acetate and isopentyl acetate were detected first (peaks 3 and 5). Lastly, *D. suzukii* first responded in GC/SSR to methyl butyrate, methyl isovalerate, and isopentyl acetate in the blush red phase of fruit development (peaks 1, 2, and 5). Moreover, using SSR stimulation at six different concentrations (from 10^−8^ to 10^−3^), we were able to show again that the “ab3A” OSN in *D. suzukii* is more sensitive than *D. melanogaster* towards several of the compounds associated with strawberry fruit (Fig. 2d). However, in subsequent behavioral trials using all developmental stages, there was only one difference in attraction noted among the three *Drosophilia* species (Green stage, Fig. 2b). In fact, when given the choice between every stage of the ripening process, all three fly species preferred the later stages of fruit development, especially the overripe and rotten stages. While more *D. suzukii* were captured than the other two fly species in traps containing green and white fruit stages, only the attraction to green fruit was significantly different among the species (Green, *P* = 0.019 and White, *P* = 0.08, respectively). Additionally, *D. melanogaster* was captured in higher numbers than *D. suzukii* in behavioral trials using fruit-related compounds, such as hexyl acetate and isopentyl acetate (Supplemental 1C).

### Attraction Towards Leaf Tissue

**Fig. 3:**
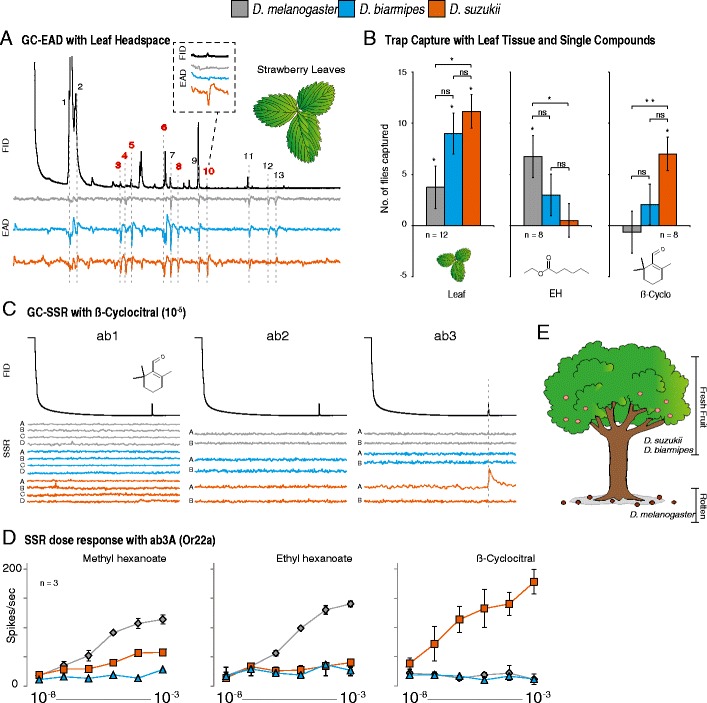
Electrophysiological and behavioral responses using leaf chemistry. (**a**) GC/coupled electroantennogram recordings (GC/EAD) with leaf headspace (strawberry) (*N* = 3). *Top graph*, GC trace (FID) of leaf headspace; *bottom graphs*, EAD responses (*Grey* = *Drosophila melanogaster*; *Blue* = *D. biarmipes*; *Orange* = *D. suzukii*). Inset figure depicts the *D. suzukii*-specific response to β-cyclocitral. GC peaks were identified (and confirmed with synthetic standards) as (*1*) Z-3-hexenol, (*2*) E-2-hexenol, (*3*) 1-octen-3-ol, (*4*) 6-methyl-5-hepten-2-ol, (*5*) Z-3-hexenyl acetate, (*6*) E-2-nonenol, (*7*) phenethyl alcohol, (*8*) 2-nitrophenol, (*9*) methyl salicylate, (*10*) β-cyclocitral, (*11*) eugenol, (*12*) β-ionone, (*13*) unknown. (**b**) Trap-capture rates of the three *Drosophila* species using either whole leaf, the fruit compound ethyl hexanoate (EH), or the leaf compound β-cyclocitral (β-Cyclo). An *asterisk* denotes significant differences at α ≤ 0.05 between the treatment and control or between the species tested, while two asterisks denote significance at α ≤ 0.01 (Two-tailed, paired *t*-test). (**c**) GC/coupled single sensillum recordings (GC/SSR) with β-cyclocitral across all large basiconic sensilla of the three *Drosophila* species (*N* = 3). (**d**) Dose response curves (SSR) for each fly species to the best “ab3A” OSN ligands for *D. melanogaster* (methyl and ethyl hexanoate), as well as to the best ligand for *D. suzukii* “ab3A” (β-cyclocitral). (**e**) Microhabitats where the three *Drosophila* species usually occur, showing separation in preference

Headspace odor collections also were generated from host plant leaves (*e.g*., Strawberry and Cherry), and using GC/EAD trials it was shown that both of the spotted-wing Drosophilids were more sensitive than *D. melanogaster* to the majority of the chemical cues associated with leaf tissue (Fig. 3a; Supplemental 2A). One of the compounds associated with the leaf tissue, β-cyclocitral, was detected only by *D. suzukii*, and thus appears to be species-specific (Fig. 3a, highlighted region). In behavioral trials, all three fly species were attracted to whole strawberry leaves when presented against a blank control, with *D. suzukii* was more attracted than *D. melanogaster* (Fig. 3b; *P* = 0.013). Using trap assays, volatile compounds identified from the leaf tissue (E-2-nonenol, 2-nitrophenol, and β-cyclocitral; peaks 6, 8, and 10, Fig. 3a) were more effective at capturing the two species of spotted-wing Drosophilids than in capturing *D. melanogaster* (Fig. 3b; Supplemental 2D), whereas *D. melanogaster* was captured more effectively with volatiles associated with ripe or overripe fruits, as well as those odors associated with fermentation (ethyl hexanoate and the combination of isopentyl acetate, butyl acetate as well as hexyl acetate) (Fig. 3b, Supplemental 2D). More specifically, we demonstrated that β-cyclocitral is attractive only to *D. suzukii*, whereas E-2-nonenol and 2-nitrophenol were attractive only to *D. biarmipes* in these behavioral trap assays, all of which were compounds identified from host plant foliage and not from the fruit. We confirmed that stressed leaves release more of several volatile compounds, including β-cyclocitral. Furthermore, when presented with a choice between intact strawberry leaves and stressed leaves (*e.g*., mechanical damage, solvent, or frost-thaw shock), *D. suzukii* showed an increased attraction towards leaf tissue that was stressed (*P* < 0.001 and *P* = 0.008, respectively) (Supplemental 2C). We also tested ethylene gas as a possible attractant at three concentrations (5, 1, and 0.1%); however, none of the *Drosophila* species showed any behavioral preference for this compound.

### β-Cyclocitral Detected by OSNs Housed Within the “ab3” Sensillum

The closest matching response profile for the *D. suzukii* OSN associated with the SSR response towards β-cyclocitral was “ab3A”, which houses the Or22a neuron in *D. melanogaster* (Fig. 1c). Here, we showed that “ab3A” in both *D. suzukii* and *D. biarmipes* has a diminished response to the fermentation odors methyl and ethyl hexanoate (the best ligands for Or22a in *D. melanogaster*), and when compared to *D. melanogaster* in behavioral trials, both spotted-wing *Drosophila* were less attracted to ethyl hexanoate (Fig. 3b). While the OSN(s) in *D. biarmipes* that are responsible for the detection of the leaf compounds E-2-nonenol and 2-nitrophenol have not yet been identified, we show that in *D. suzukii* the “ab3A” neuron is responsible for the detection of β-cyclocitral, a novel ligand associated with the leaf tissue of its host plants (Fig. 3e).

## Discussion

While the majority of the *Drosophila* species within the *melanogaster* clade have been shown to be most attracted by the fermentation byproducts of decaying fruit material as well as from the associated yeast, several other feeding and oviposition niches have been documented within the family Drosophilidae, including several species of fly that are ecologically-bound to leaf tissue. A prime example of this ecological specialization is *Scaptomyza flava* and *S. nigrita*, both of which are members of an herbivorous leaf-mining lineage within Drosophilidae, where adult females use their sclerotized ovipositor to puncture the leaf surface in order to feed and lay eggs within their host plant (Whiteman et al., 2011). Perhaps, as an evolutionary intermediate host between rotten fruit and living leaves, some Drosophilids are attracted to fermenting plant or leaf tissue, such as the ecological system of *D. mojavensis*, a group of flies that specializes on fermenting cactus photosynthetic tissue, as opposed to specializing on the fruit of its host (Date et al., 2013). It also has been shown that some *Drosophila* are attracted to tree sap, such as *D. virilis* (Carson and Stalker, 1951) as well as *D. pseudoobscura* (Dobzhansky and Queal, 1938), or have been observed to feed on leaves within the canopy, such as *D. obscura* and *D. subobscura* (Begon, 1975; Shorrocks 1975).

Thus, the *D. suzukii* association with leaf volatiles that we document here is not the first reported case of this type of behavioral adaptation within Drosophilidae. However, it is new in the regard that the attractive volatiles do not emanate directly from the oviposition source itself, but rather they may serve as a signal of the existence of ripening fruits nearby. This is supported by the fact that we show that developing fruit do not produce dramatic olfactory cues that are detected by *D. suzukii* until the onset of the blush red phase of fruit development (GC/EAD data not shown). Although the three species differ in their sensitivity regarding the detection of fruit-ripening dependent volatiles, with *D. suzukii* and *D. biarmipes* being more sensitive than *D. melanogaster*, this difference is not reflected in any species-specific behavioral preference towards different ripening stages of the fruit, nor does the fruit odor alone explain the preference of *D. suzukii* to attack fresh, as opposed to overripe or rotten substrates. However, the attraction towards leaf tissue likely explains their reported presence in the plant canopy during the developmental stages of the fruit (Mitsui et al., 2006; Poyet et al., 2014), and it also is probably linked to the subsequent attack on fresh fruit by *D. suzukii* for feeding and oviposition. The olfactory sensitivity of *D. suzukii* towards leaf tissue likely plays a role in this fly species identifying and attacking early stages of fruit ripening, perhaps due to an increased proximity of the adult flies within the foliage or canopy, prior to or during fruit ripening stages, that are suitable for feeding and oviposition (Fig. 3e). In addition, several publications already have demonstrated that *D. suzukii* is more likely to oviposit on fruit that is within the leaf canopy of the host plant, as opposed to fallen fruit that is separated from the leaves (Mitsui et al., 2006; Poyet et al., 2014).

This is in contrast to *D. melanogaster*, which has been shown repeatedly to prefer fermenting or rotten fruits, and moreover does not possess the sclerotized ovipositor necessary to puncture fresh, ripe fruits. Additionally, it has been suggested that in blackberry, raspberry, and strawberry plants the stage of fruit development might alter the nearby leaf volatile chemistry (El Hadi et al., 2013; Wang and Lin, 2000), perhaps due to the stress of fruit development, which may further provide navigational cues to *D. suzukii* adults that are seeking young fruit that is suitable for feeding or oviposition (Supplemental 2C). Moreover, this olfactory sensitivity to leaf chemistry in *D. suzukii* appears to be regulated by the “ab3A” neuron, an OSN that has been shown repeatedly to play a role in species differences for feeding and oviposition preference. Previously, the “ab3A” neuron has been shown to regulate host plant preference towards a toxic fruit niche for *D. sechellia* (Dekker et al., 2006), and again in the preference of *D. erecta* towards egg-laying upon *Pandanus* fruit (Linz et al., 2013). It also has been demonstrated that the blush phase of strawberry development is the first stage that displays dramatic color change (from green or white to bright red). Therefore, it also may be important to address visual differences in *D. suzukii* that further aid in this species locating fresh fruit within the leaf canopy of host plants, as vision also is important in trapping this species (Basoalto et al., 2013). It also should be noted that the compound β-cyclocitral often is associated with algae or yeast (Jüttner et al., 2010; Mendes-Pinto, 2009); however, whether β-cyclocitral was produced directly by the plant or instead by an associated microbial organism remains unclear, although the compound has been found previously from volatile collections of strawberries (El Hadi et al., 2013). Nonetheless, it may be important to test leaf-associated microbial strains for the production of additional compounds that *D. suzukii* might be highly attracted towards. Further work also is necessary to ascertain whether the combination of leaf and fruit odors will maximize *D. suzukii* capture, and additional studies are required to determine optimal concentrations of β-cyclocitral for field testing or subsequent monitoring efforts.

## Electronic supplementary material

Below is the link to the electronic supplementary material.

Supplemental 1 Electrophysiological and behavioral responses towards fruit developmental stages and the associated volatile chemistry. (A) Dose response curves (SSR) for the “ab2B” OSN towards several compounds identified from strawberry fruit headspace that *Drosophila suzukii* was shown to be more sensitive to than *D. melanogaster* in GC/SSR trials. (B) GC/coupled single sensillum recordings (“ab2” sensillum) using headspace samples from eight distinct stages of fruit development. Headspace collections are shown above (FID) with the respective A and B neuron response for each species shown below (SSR). (Grey = *D. melanogaster*; Blue = *D. biarmipes*; Orange = *D. suzukii*). Peaks were identified as (1) butyl acetate, (2) isopentyl acetate, (3) unknown, (4) hexyl acetate, (5) unknown, (6) unknown. (C) Trap-capture rates of the *D. melanogaster* and *D. suzukii* using identified fruit compounds (*N* = 6). An asterisk denotes a significant difference between the treatment and control or between the species tested (α = 0.05, two-tailed, paired *t*-test, GraphPad InStat version 3.10). Note that no compound was more attractive to *D. suzukii* than *D. melanogaster* in these trials. Therefore fruit odors, while attractive to *D. suzukii*, do not lend themselves towards a species-specific monitoring tool when used alone. (PDF 3058 kb)

Supplemental 2 Behavior and electrophysiological responses of the tested Drosophila species towards leaf chemistry. (A) GC/coupled electroantennogram recordings with leaf headspace (cherry). Inset figure depicts *D. suzukii*-specific response to β-cyclocitral. Top graph, GC trace (FID) of leaf headspace; bottom graphs, EAD responses; Grey = *Drosophila melanogaster*; Blue = *D. biarmipes*; Orange = *D. suzukii*. GC peaks were identified (and confirmed with synthetic standards) as (1) Z-3-hexenol, (2) E-2-hexenol, (3) 1-octen-3-ol, (6) E-2-nonenol, (7) phenethyl alcohol, (8) 2-nitrophenol, (10) β-cyclocitral, (12) β-ionone, (13) unknown. (B) In addition to cherry and strawberry leaves, we also tested several other plant species, all of which contained β-cyclocitral (data not shown), though in rather variable quantities. Thus, it appears this compound is rather ubiquitous to all leaf tissue, across both potential host and non-host plants. (C) Trap-capture rates of the three *Drosophila* species comparing damaged with undamaged leaves. (e.g., solvent or frost-thaw shock). Note that damage increases the attractiveness for *D. suzukii* and increases the amount of released β-cyclocitral. (D) Trap-capture rates of the three *Drosophila* with fruit and leaf compounds. Behavioral trials were conducted using compounds from leaf tissue that the spotted-wing *Drosophila* were more sensitive towards in GC/EAD recordings. We also tested a combination of the fruit odors that the spotted-wing *Drosophila* were shown to be more sensitive towards (Butyl acetate, isopentyl acetate and Hexyl acetate); however, once again *D. melanogaster* was more attracted than the other two species to this combination of fruit odors (PDF 2835 kb)
