# Supplementary figures and images for: Olfactory Specialization in Drosophila suzukii Supports an Ecological Shift in Host Preference from Rotten to Fresh Fruit
Source: J Chem Ecol. 2015 Jan 25;41(2):121–8. doi: 10.1007/s10886-015-0544-3 (PMC4351439; doi:10.1007/s10886-015-0544-3)

A ab2B

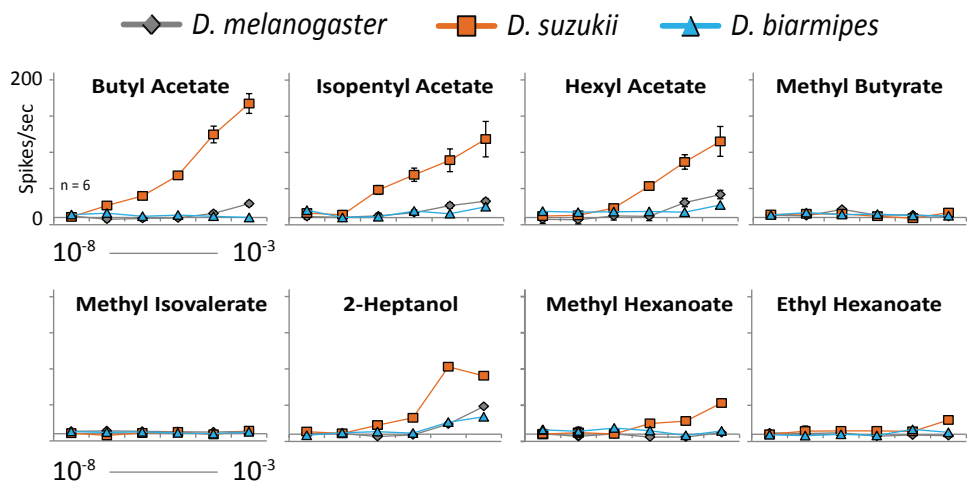

B GC-SSR (ab2)

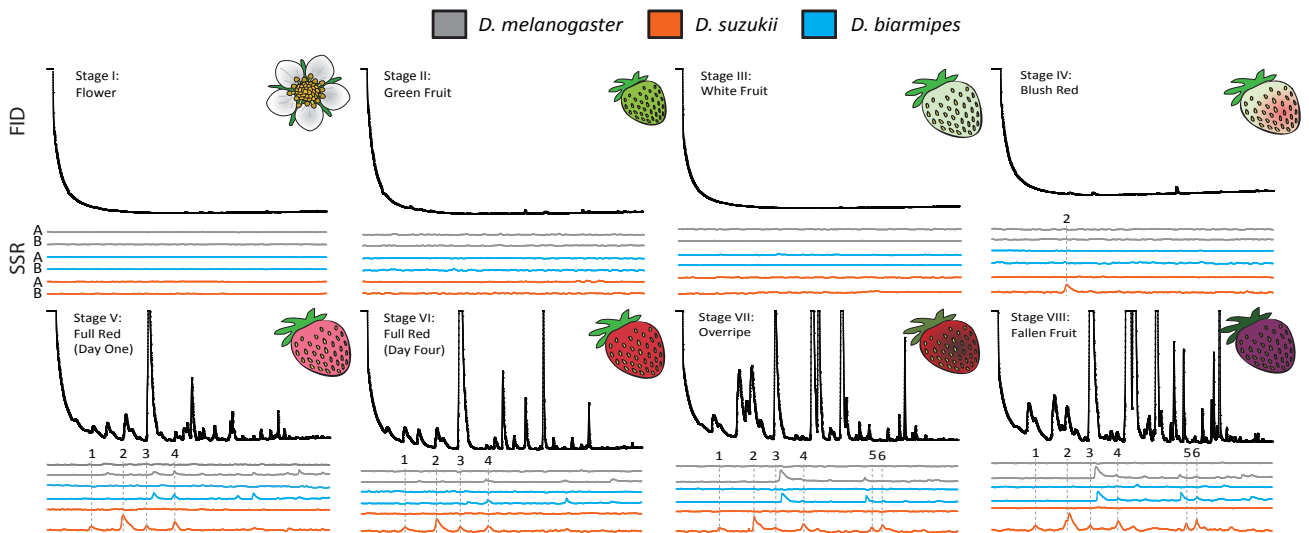

C Trap assay with fruit compounds

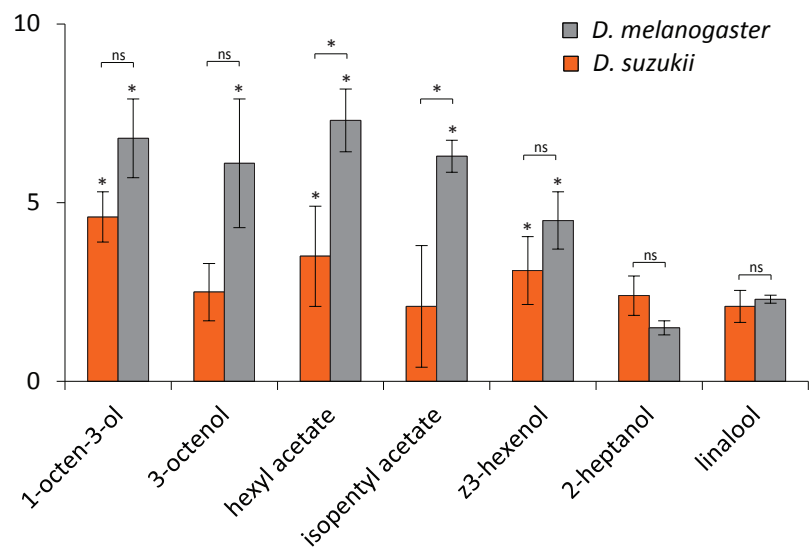

Supplement: Supplementary file 1 — Electrophysiological and behavioral responses towards fruit developmental stages and the associated volatile chemistry. (A) Dose response curves (SSR) for the “ab2B” OSN towards several compounds identified from strawberry fruit headspace that Drosophila suzukii was shown to be more sensitive to than D. melanogaster in GC/SSR trials. (B) GC/coupled single sensillum recordings (“ab2” sensillum) using headspace samples from eight distinct stages of fruit development. Headspace collections are shown above (FID) with the respective A and B neuron response for each species shown below (SSR). (Grey = D. melanogaster; Blue = D. biarmipes; Orange = D. suzukii). Peaks were identified as (1) butyl acetate, (2) isopentyl acetate, (3) unknown, (4) hexyl acetate, (5) unknown, (6) unknown. (C) Trap-capture rates of the D. melanogaster and D. suzukii using identified fruit compounds (N = 6). An asterisk denotes a significant difference between the treatment and control or between the species tested (α = 0.05, two-tailed, paired t-test, GraphPad InStat version 3.10). Note that no compound was more attractive to D. suzukii than D. melanogaster in these trials. Therefore fruit odors, while attractive to D. suzukii, do not lend themselves towards a species-specific monitoring tool when used alone. (PDF 3058 kb) [file 10886_2015_544_MOESM1_ESM.pdf]
