# Supplementary material for: Olfactory Specialization in Drosophila suzukii Supports an Ecological Shift in Host Preference from Rotten to Fresh Fruit
Source: J Chem Ecol. 2015 Jan 25;41(2):121–8. doi: 10.1007/s10886-015-0544-3 (PMC4351439; doi:10.1007/s10886-015-0544-3)
Supplement: Supplementary file 2 — Behavior and electrophysiological responses of the tested Drosophila species towards leaf chemistry. (A) GC/coupled electroantennogram recordings with leaf headspace (cherry). Inset figure depicts D. suzukii-specific response to β-cyclocitral. Top graph, GC trace (FID) of leaf headspace; bottom graphs, EAD responses; Grey = Drosophila melanogaster; Blue = D. biarmipes; Orange = D. suzukii. GC peaks were identified (and confirmed with synthetic standards) as (1) Z-3-hexenol, (2) E-2-hexenol, (3) 1-octen-3-ol, (6) E-2-nonenol, (7) phenethyl alcohol, (8) 2-nitrophenol, (10) β-cyclocitral, (12) β-ionone, (13) unknown. (B) In addition to cherry and strawberry leaves, we also tested several other plant species, all of which contained β-cyclocitral (data not shown), though in rather variable quantities. Thus, it appears this compound is rather ubiquitous to all leaf tissue, across both potential host and non-host plants. (C) Trap-capture rates of the three Drosophila species comparing damaged with undamaged leaves. (e.g., solvent or frost-thaw shock). Note that damage increases the attractiveness for D. suzukii and increases the amount of released β-cyclocitral. (D) Trap-capture rates of the three Drosophila with fruit and leaf compounds. Behavioral trials were conducted using compounds from leaf tissue that the spotted-wing Drosophila were more sensitive towards in GC/EAD recordings. We also tested a combination of the fruit odors that the spotted-wing Drosophila were shown to be more sensitive towards (Butyl acetate, isopentyl acetate and Hexyl acetate); however, once again D. melanogaster was more attracted than the other two species to this combination of fruit odors (PDF 2835 kb) [file 10886_2015_544_MOESM2_ESM.pdf]

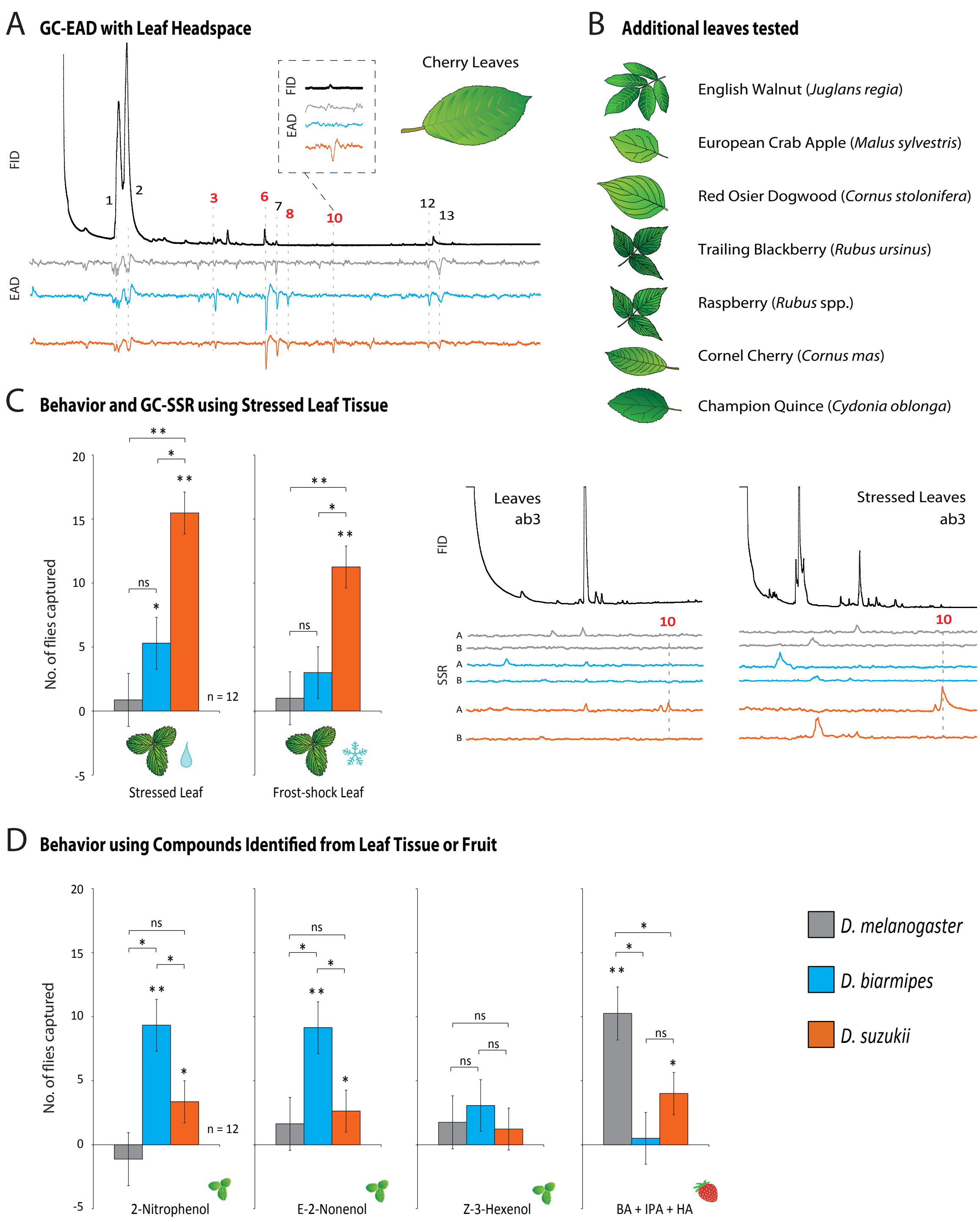
A GC-EAD with Leaf Headspace
FID
EAD
Cherry Leaves
1
2
3
6
7
8
10
12
13
B Additional leaves tested
English Walnut (Juglans regia)
European Crab Apple (Malus sylvestris)
Red Osier Dogwood (Cornus stolonifera)
Trailing Blackberry (Rubus ursinus)
Raspberry (Rubus spp.)
Cornel Cherry (Cornus mas)
Champion Quince (Cydonia oblonga)
C Behavior and GC-SSR using Stressed Leaf Tissue
No. of flies captured
n = 12
ns
Stressed Leaf
Frost-shock Leaf
Leaves ab3
Stressed Leaves ab3
SSR
D Behavior using Compounds Identified from Leaf Tissue or Fruit
2-Nitrophenol
E-2-Nonenol
Z-3-Hexenol
BA + IPA + HA
D. melanogaster
D. biarmipes
D. suzukii
